# Contextual factors influencing the implementation of innovations in community-based primary health care: the experience of 12 Canadian research teams

**DOI:** 10.1017/S1463423619000483

**Published:** 2019-06-28

**Authors:** Jenny Ploeg, Sabrina T. Wong, Kasra Hassani, Marie-Lee Yous, Martin Fortin, Claire Kendall, Clare Liddy, Maureen Markle-Reid, Bojana Petrovic, Emilie Dionne, Cathie M. Scott, Walter P. Wodchis

**Affiliations:** 1 Department of Health, Aging and Society, School of Nursing, Scientific Director, Aging, Community and Health Research Unit, Faculty of Health Sciences and Associate Member, McMaster University, Hamilton, ON, Canada; 2 School of Nursing and Director, Centre for Health Services and Policy Research, University of British Columbia, Vancouver, BC, Canada; 3 School of Nursing and Centre for Health Services and Policy Research, University of British Columbia, Vancouver, BC, Canada; 4 School of Nursing, McMaster University, Hamilton, ON, Canada; 5 Department of Family Medicine, Université de Sherbrooke, Sherbrooke, QC, Canada; 6 C.T. Lamont Primary Healthcare Research Group, Bruyère Research Institute, Ottawa, ON, Canada; 7 Department of Family Medicine, University of Ottawa, Ottawa, ON, Canada; 8 Department of Health Research Methods, Evidence, and Impact, Faculty of Health Sciences, Aging, Community and Health Research Unit, School of Nursing and Associate Member, McMaster University, Hamilton, ON, Canada; 9 PhD Student, Department of Family and Community Medicine, Dalla Lana School of Public Health, University of Toronto, Toronto, ON, Canada; 10 St. Mary’s Research Centre, St. Mary’s Hospital, Montréal, QC, Canada; 11 Department of Community Health Sciences & Sociology, University of Calgary, Calgary, AB, Canada; 12 Institute of Health Policy, Management and Evaluation, Research Chair in Implementation and Evaluation Science, University of Toronto, Toronto, ON, Canada; 13 Institute for Better Health, Trillium Health Partners, University of Toronto, Toronto, ON, Canada

**Keywords:** context, health care innovations, implementation, primary care

## Abstract

The objectives of this paper are to: (1) identify contextual factors such as policy that impacted the implementation of community-based primary health care (CBPHC) innovations among 12 Canadian research teams and (2) describe strategies used by the teams to address contextual factors influencing implementation of CBPHC innovations. In primary care settings, consideration of contextual factors when implementing change has been recognized as critically important to success. However, contextual factors are rarely recorded, analyzed or considered when implementing change. The lack of consideration of contextual factors has negative implications not only for successfully implementing primary health care (PHC) innovations, but also for their sustainability and scalability. For this evaluation, data collection was conducted using self-administered questionnaires and follow-up telephone interviews with team representatives. We used a combination of directed and conventional content analysis approaches to analyze the questionnaire and interview data. Representatives from all 12 teams completed the questionnaire and 11 teams participated in the interviews; 40 individuals participated in this evaluation. Four themes representing contextual factors that impacted the implementation of CBPHC innovations were identified: (I) diversity of jurisdictions (II) complexity of interactions and collaborations (III) policy, and (IV) the multifaceted nature of PHC. The teams used six strategies to address these contextual factors including: (1) conduct an environmental scan at the beginning (2) maintaining engagement among partners and stakeholders by encouraging open and inclusive communication; (3) contextualizing the innovation for different settings; (4) anticipating and addressing changes, delays, and the need for additional resources; (5) fostering a culture of research and innovation among partners and stakeholders; and (6) ensuring information about the innovation is widely available. Implementing CBPHC innovations across jurisdictions is complex and involves navigating through multiple contextual factors. Awareness of the dynamic nature of context should be considered when implementing innovations.

## Introduction

Contextual factors, broadly defined as all intervening factors that affect a complex phenomenon (Stange *et al*., 2012; Bate *et al*., 2014), are recognized as critical to successful health care delivery and implementation of health care innovations (Ovretveit, 2011; Evans *et al*., 2017; Pfadenhauer *et al*., 2017; Abuzour *et al*., 2018). Examples of contextual factors are: (a) the external context (eg, policy and legislation, buy-in by internal or external stakeholders) (b) the organization (eg, organizational culture, leadership, resources, and relationships) (c) professionals (eg, roles, competency); and (d) intervention (eg, nature and characteristics, complexity) (Damschroder *et al*., 2009; Lau *et al*., 2016). In primary care settings, consideration of contextual factors when implementing health care innovations is recognized as a strategy for maximizing uptake and success (Lau *et al*., 2016).

Innovation in primary care consists of implementing creative solutions to provide high quality care for clients through efficient and strategic use of available resources (eg, funding and staff) (Okie, 2008). The lack of consideration of contextual factors has negative implications for successfully implementing health care innovations, sustainability, and scalability (Stirman *et al*., 2012; Milat *et al*., 2015). However, contextual factors are rarely recorded, analyzed or considered when implementing change (Tomoaia-Cotisel *et al*., 2013). Thus, attempts to implement or transfer innovations to other settings are often unsuccessful, because contextual factors are poorly integrated into change efforts (Shekelle *et al*., 2010; Kringos *et al*., 2015; May *et al*., 2016).

Previous work has explored the influence of contextual factors on change in primary care settings (Carlfjord *et al*., 2010; Tierney *et al*., 2014; Shea *et al*., 2018; Ward *et al*., 2018). This work suggests a number of contextual factors as important to consider for successful implementation of health care innovations, such as: teamwork climate (Shea *et al*., 2018), organizational capacity to take on new initiatives (Carlfjord *et al*., 2010; Shea *et al*., 2018), objectives and perceived value of the innovation (Shea *et al*., 2018; Ward *et al*., 2018), and top-level endorsement (Tierney *et al*., 2014).

Tomoaia-Cotisel *et al*. (2013) reported on contextual factors across 14 studies of primary care practice transformation to patient-centered medical homes (PCMHs). Using results from these 14 studies, they developed a novel framework for reporting contextual factors, based on the work of Stange and Glasgow (2013). Stange and Glasgow’s (2013) template includes the following contextual factors: national, provincial and local policy, community norms, organization of health care, practice culture, and historical factors. Tomoaia-Cotisel *et al.’s* (2013) framework expands on the template and describes contextual factors in three hierarchical dimensions (practice, larger organization, and external environment) and two cross-cutting dimensions (implementation pathway and motivations). While this paper provides a valuable framework that can be used to report contextual factors in primary care change initiatives, it is focused on one specific type of transformation, that of PCMHs, and does not describe strategies that could be used to address these contextual factors.

The purposes of this paper are to: (1) describe contextual factors that impacted the implementation of community-based primary health care (CBPHC) innovations among 12 Canadian research teams and (2) describe strategies used by the teams to address contextual factors influencing implementation of CBPHC innovations. In 2012, as part of a signature initiative in CBPHC research, the Canadian Institutes of Health Research (CIHR) funded 12 pan-Canadian research teams (hereafter referred to as 12 teams) to conduct five-year programmatic cross-jurisdictional innovative research projects (CIHR, 2012a; CIHR, 2013). The funding’s key aims included: (a) reduction of inequities in access to CBPHC and health outcomes of vulnerable populations and (b) prevention and management of chronic conditions (CIHR 2012b).

Teams were also required to collaborate and produce knowledge beyond what could be produced by any one team, including collection of data on a common set of patient and practice indicators (Wong *et al*., 2018) and description of the structures and context that influence the implementation and potential for scale-up of successful CBPHC innovations (Ben Charif *et al*., 2018). The 12 teams are composed of researchers, trainees, patient and community research partners and decision makers. The teams are located within rural and urban settings in multiple provinces across Canada (eg, Alberta, British Columbia, Manitoba, New Brunswick, Newfoundland and Labrador, Nova Scotia, Northwest Territories, Nunavut, Ontario, Prince Edward Island, and Quebec) and some teams included international partners (eg, Australia, Belgium, Denmark, France, New Zealand, Philippines, Singapore, and the United States). The areas of focus are varied and include, for example, prevention and management of chronic conditions and access to care for vulnerable groups. These teams are implementing innovative projects to improve CBPHC for diverse populations such as children, youth, older adults, immigrants, low-income groups and Indigenous Peoples. The teams have varied goals such as using technology to improve HIV care and self-management, using patient experiences in reshaping primary health care (PHC) to address the needs of First Nation communities, and partnering with local community health and social care providers to improve care for older adults with diabetes and multimorbidity. More detail about the 12 teams is available on the CIHR website: http://www.cihr-irsc.gc.ca/e/50370.html.

An example of one of the teams and their innovations follows. **Canadian Team to Improve Community-Based Cancer Care along the Continuum (**CanIMPACT) is a multi-disciplinary team of primary care providers, cancer specialists, researchers, patients and caregivers who are working together to improve care coordination for patients with cancer. In collaboration with the Champlain BASE™ eConsult team, CanIMPACT is evaluating an asynchronous online communication system (“eOncoNote”) that aims to improve communication between primary care providers and cancer specialists (Grunfeld, 2017; Grunfeld and Petrovic, 2017).

## Methods

### Design

A qualitative evaluation approach was used.

### Data collection

Data collection was conducted using self-administered questionnaires and follow-up telephone interviews with representatives of the 12 teams, from May to December 2017. Two authors (KH and JP) developed a questionnaire adapted from Stange and Glasgow’s Context Matters Reporting Template (2013), and Tomoaia-Cotisel *et al*.’s (2013) framework of contextual factors in PHC. The questionnaire was piloted with the co-leads of one of the 12 teams to assess face validity (ie, questions were clear and relevant). The questionnaire asked teams to describe their project, study population, and partnering stakeholders (eg, clinicians, community members, and decision makers). Teams were then asked to describe contextual factors impacting the implementation of their innovations, regional public policy, and multi-jurisdictional nature of the work (See Supplementary material – Appendix 1 for survey questions).

Follow-up semi-structured individual and group telephone interviews, lasting 30–45 min, were conducted by two authors (KH and JP) with each team. One to three persons from each team were interviewed, including researchers and research staff (research coordinators and managers). Participants were asked to identify the contextual factors that had been most influential in the implementation of their innovation, how these factors had influenced their work, and strategies they used to address contextual factors. Additional probing questions were asked to clarify responses to the questionnaires. Interviews were audio-recorded and transcribed (See Supplementary material – Appendix 2 for sample interview questions).

### Data analysis

We used a combination of directed and conventional content analysis approaches (Hsieh and Shannon, 2005) to analyze the questionnaire and interview data. Using a directed content analysis approach, we started with Tomoaia-Cotisel *et al*.’s (2013) framework to develop general codes and categories of the contextual factors that impacted the implementation of CBPHC innovations. Building on this framework, we then used a conventional content analysis approach where additional codes and categories were derived directly from the questionnaire and interview texts.

Three authors (KH, JP and MY) independently reviewed the questionnaire and interview data and developed initial codes and categories. These authors met monthly over four months to discuss and compare codes and categories and develop a final coding structure. Two authors (KH and MY) applied the final coding scheme to the data. Coded data were reviewed and themes and sub-themes were identified by consensus and reviewed by the larger research team. We used N-Vivo 11® (QSR International) to assist with qualitative data analysis.

We ensured rigor by using Lincoln and Guba’s (1985) validation criteria (ie, credibility, transferability, dependability, and confirmability). Credibility was established by seeking a broad range of participants (team leads, project managers, and research coordinators) and using investigator triangulation. In addition to the three primary data analysts (KH, JP, and MY), nine co-authors (SW, MF, CK, CL, MMR, BP, ED, CS, and WPW) with expertise in program evaluation, CBPHC, and qualitative research provided feedback on the themes to ensure that they were accurate and reflective of their experiences. Descriptions of the teams and their innovations help to ensure transferability of findings (Creswell, 2017). Dependability and confirmability were achieved by maintaining a record of all analytic decisions.

### Ethics statement

All procedures contributing to this work comply with the ethical standards of the Tri-Council Policy Statement, Ethical Conduct for Research Involving Humans.

## Results

### Participants

Representatives from all 12 teams completed the questionnaire and 11 teams participated in the interviews (one team was not available for an interview). A total of 40 people participated in the data collection, of whom 29 (72%) were senior and junior researchers (principal investigators, collaborators, and postdoctoral researchers) and 11 (28%) were research staff (research managers and research coordinators). Between 1 and 6 people per team participated in the data collection (median = 3). The representatives were diverse in terms of gender, age, years of research experience, and work locations. Table 1 provides a brief summary of the 12 teams including location, study population, area of focus and research or innovation project.

**Table 1.** Overview of the 12 CBPHC teams

Nominated principal investigator (Project Name)	Location(s)	Study population	Area(s) of focus	Research and/or innovation project
Marshall Godwin (Atlantic Canada Children’s Effective Service Strategies -Mental Health (ACCESS-MH))	Atlantic Canada (ie, Prince Edward Island, New Brunswick, Nova Scotia, and Newfoundland) International Partner in New Zealand	Children and youth, parents, and service providers	Autism spectrum, conduct, and eating disorders among children and youth	Assuming a broad social sciences approach for each condition using Patient Journeys/Process Mapping, Operations Research and Statistical analysis of complex databases to understand and explore how children and youth access treatment and services across multiple health systems such as the health and education systems
Eva Grunfeld (Canadian Team to Improve Community-Based Cancer Care along the Continuum (CanIMPACT))	British Columbia, Alberta, Manitoba, Ontario, Nova Scotia, New Brunswick, and Newfoundland and Labrador International partners in the United States, Australia, and Denmark	Breast and colorectal cancer patients and health care providers	Cancer journey with a special focus on breast cancer for vulnerable populations (ie, older adults, individuals living in rural, remote or northern areas, low- income groups, and immigrants)	Exploring multiple perspectives in the cancer journey, conducting an environmental scan to enhance integration of care between primary care and cancer specialist care, and developing and testing an intervention to improve care coordination for patients with cancer (Grunfeld, 2017; Grunfeld and Petrovic, 2017)
Jeannie Haggerty (Innovative Models Promoting Access-to-Care Transformation (IMPACT))	Quebec, Ontario, and Alberta International Partners in Australia (ie, New South Wales, South Australia, and Victoria)	Socially vulnerable patients living in highly deprived neighbourhoods who have low language proficiency, social support, health literacy, or used emergency services within the past year for minor treatment	Access to care for vulnerable populations	Identifying and evaluating primary health care-based innovations to improve access to primary health care for vulnerable groups and determining the effectiveness and scalability of innovations
Stewart Harris (TransFORmation of IndiGEnous PrimAry HEAlthcare Delivery (FORGE AHEAD))	British Columbia, Alberta, Manitoba, Ontario, Quebec, and Newfoundland	First Nations communities	Chronic disease management (ie, type two diabetes mellitus)	Developing and evaluating community-inspired and culturally relevant primary health care models to improve access to services as well as enhancing the spread of successful innovations for First Nations communities in Canada
Janusz Kaczorowski (The Canadian Chronic Disease and Awareness Program (C-ChAMP))	Ontario, Alberta, and Quebec International partner in the Philippines	Vulnerable populations, French-speaking communities, South Asian communities, and patients without a family physician	Prevention and management of chronic disease among various populations and settings with a particular focus on cardiovascular health	In follow-up to the success of the Cardiovascular Health Awareness Program (CHAP), a program called Canadian Chronic Disease Awareness and Management Program (C-ChAMP) has been developed to be applied among various populations and settings and optimal conditions for implementation will be identified to sustain the project Canada-wide and internationally
Alan Katz (Innovation Supporting Transformation in Community-Based Primary Healthcare Research Project (iPHIT))	Rural and remote Manitoba First Nations	Northern and Southern First Nation communities in Manitoba	Primary health care within First Nations communities	Transforming primary health care through the perspectives and suggestions for innovations of First Nation communities based on their health and needs
Claire Kendall (Living with HIV (LHIV) Innovation Team)	Manitoba, Ontario, Newfoundland, and Labrador	People living with HIV	HIV care within community-based settings	Improving HIV care by implementing eHealth technologies, enhancing the patient experience and self-management and recognizing necessary measures to promote a more integrated primary care model
Jenny Ploeg, Maureen Markle-Reid (Aging, Community, Health and Research Unit (ACHRU)	Alberta and Ontario	Older adults with multiple chronic conditions, family caregivers, and health care professionals	Multiple chronic conditions and type two diabetes mellitus among community-based older adults	Community Partnership Program for older adults with diabetes and multimorbidity (Markle-Reid *et al*., 2018).
Moira Stewart (Patient Centred Innovations for Persons with Multimorbidity (PACE in MM))	Ontario, Quebec, British Columbia, Manitoba, Nova Scotia, New Brunswick International Partners in Belgium, France, Argentina, and Singapore	Patients with multi-morbidities aged 18–80	Chronic disease prevention and management	Identifying responsible factors for the success or failure of current initiative in chronic disease care, testing and comparing new innovations in two or more jurisdictions, and promoting scaling up of innovations
Walter Wodchis (implementing integrated Care for Older Adults with Complex Health needs (iCOACH))	Quebec and Ontario International partner in New Zealand	Chinese communities, low-income seniors, French-speaking communities, clinically complex groups, and populations in Maori (ie, Indigenous and rural, mixed Maori and European, and primarily European)	Scaling up successful intervention in primary health care for older adults with complex needs	Designing and implementing innovative models in primary health care for older adults with complex needs across three jurisdictions and evaluate the contextual factors that influence the success or failure of the programs
Sabrina Wong (Transforming Community-Based Primary Healthcare through Comprehensive measurement and reporting (TRANSFORMATION)	British Columbia, Ontario and Nova Scotia	Organizational leads, health care providers, and patients from family physician practices	Community-based primary health care performance measurement and health care equity	Developing a comprehensive primary health care-based framework for performance measurement and reporting of contextual information related to the spread and uptake of primary health care innovations and strategies
Kue Young (The Circumpolar Health System Innovation Team (CircHSIT)	The Northwest Territories, Nunavut, and Labrador	Aboriginal and non-Aboriginal groups	Primary health care in remote northern communities	Transforming primary health care in remote northern communities by designing, implementing, and evaluating health systems intervention and innovations in technology

See Canadian Institutes of Health Research website for more information on the teams http://www.cihr-irsc.gc.ca/e/50370.html.

### Contextual factors

We categorized the contextual factors that impacted the implementation of CBPHC innovations of the 12 teams into four main themes with related sub-themes: (I) diversity of jurisdictions (II) complexity of interactions and collaborations (III) policy, and (IV) the multifaceted nature of PHC (See Table 2 for a summary of themes and sub-themes). Descriptions of the themes and sample quotations from interviews and questionnaires are provided below. Additional quotes are displayed in Table 3 to provide rich examples from the 12 teams.

**Table 2.** Summary of contextual factors influencing CBPHC innovations of the 12 teams and strategies to address these factors

Contextual factors	Strategies
**Theme I: Diversity of jurisdictions**	Conducting an environmental scan at the beginning
Cross-jurisdictional nature of the work	Maintaining engagement among partners and stakeholders by encouraging open and inclusive communication
International partnerships	Contextualizing the innovation for different settings
Multiple languages	Anticipating and addressing changes, delays, and the need for additional resources
**Theme II: Complexity of interactions and collaborations**	Fostering a culture of research and innovation among partners and stakeholders
Establishing and maintaining relationships	Ensuring information about the innovation is widely available
Complexity of being part of the CBPHC 12 Teams	
Level of engagement with stakeholders	
Leadership	
Working with Indigenous Peoples	
**Theme III: Policy**	
Differences in provincial health care systems	
Funding models	
Changes in legislation and health care priorities	
**Theme IV: The multifaceted nature of PHC**	
The fragmented structure of PHC	
Competing priorities	

**Table 3.** Additional examples of data for contextual factors influencing implementation of CBPHC innovations by theme

**Diversity of jurisdictions**
**1a. Cross-jurisdictional nature of the work**
*“I think what we found in our program is that we’re doing it across 7 provinces with 11 communities, with the limited amount of resources that they have, and also the amount of resources that we can put in based on our funding constraints, so we tried to come up with some creative ways to make sure that the program was as participatory…”* (T01 interview)
*“…the pieces there that were again difficult were the ethics approvals we had to get in the four jurisdictions and then not only within the four universities but also within health authorities, within education regions within the four [jurisdiction] province.”* (T09 interview)
**1b. International partners**
*“So the international collaborators, there’s…two people from the [country], two people from [country] and one person from [country]… So I will say that one of the things that facilitated the development of the protocol, linking as a group, and connecting with these international liaisons is an organization called [name] which is an international organization focusing on primary care, researchers and practitioners who are interested specifically in [chronic disease]…There were maybe 3 people in the world that were doing this.”* (T12 interview)
*“…he [research member] is also working with children and youth in [country] in the work that he is doing and often [country] and [country] are ahead of the curve in mental health issues. So, he’s been very much sending us things and bringing back ideas. Again, he’s always on our meetings every second week.”* (T09 interview)
**1c. Multiple languages**
*“Language issues give rise to other problems, such as over-reliance on relatives and ethnic staff with the right language capacities, inconsistencies in deciding which services require interpretation and the patience of the staff in dealing with a non-English speaker. There are also culturally sensitive issues that may affect immigrants, regardless of their language capacity.”* (T07 interview)
*“…we’re the only bilingual team. We’re the only team that started at the beginning with a philosophy that was we are working on the things that we have in common. We can express those things in either language because someone will translate if needed…”* (T11 interview)
**Complexity of Interactions and Collaboration**
**2a. Establishing and maintaining relationships**
*“…in every project I’ve ever worked on involving government people is the change of personnel and the inconsistency of who you deal with. It’s very hard to build relationships when you build a relationship with someone and they leave. And that turnover is such a huge issue.”* (T08 interview)
*“People’s position within the [health care network] changed, they might still be employees but they moved from a clinical role to an administrative role or…went back to school to upgrade their education.”* (T06 interview)
*“I think the point about overcoming conflict has to do with establishing those relationships [with providers] …”* (T11 interview)
*“…other researchers that just left because they took positions in other universities and institutions and didn’t feel it was relevant to stay involved in the project.”* (T05 interview)
**2b. Complexity of being part of the CBPHC 12 teams**
*“At different meetings we would have as a research team it would be reflected on well we need to keep you aware that there are 12 teams… I think it’s nice to think that you’re part of something bigger and I think that to learn things across 12 teams definitely has the potential to have some powerful outcomes.”* (T06 interview)
*“So I think it’s kind of an opportunity to spawn, among the 12 teams, relationships and other working connections beyond just at the 12 team level, but a little bit closer with regards to what the work is of the intervention we’re planning.”* (T12 interview)
*“I have always maintained the 12 teams are very different, and that although there are some cross-cutting things….I can participate in that, but a lot of other things is not that relevant…on a personal level, knowing the other teams is important. In terms of impact on the research design, the research was designed before the teams were formed.”* (T04 interview)
**2c. Level of engagement with stakeholders**
*“The status and buy-in of the leaders of the three case organizations here, at least in [province] were also impressive. The leaders of these organizations thought this was an important project, and at least a few of them are considered thought leaders in the broader health care community….”* (T10 questionnaire)
*“We had identified policy makers at the beginning of the grant but, had minimal involvement throughout. We struggled with knowing which policy makers to reach out to, along with what organizations should be involved”* (T08 questionnaire).
**2d. Leadership**
*“…leadership can come from a variety of different levels…it could be community members who are really pushing for change and improvement…it’s an organization like a tribal council or regional health authorities who recognize that they want to do things differently than what has been done…”* (T01 interview)
*“…if you’re going to do a large-scale study across multiple jurisdictions and promise a lot of high-quality work, it’s just always a lot more money and time and waiting…If you have good people in leadership, like our group it’s do-able.”* (T02 interview)
**2e. Working with Indigenous Peoples**
*“…because of our governance model and our being a First Nations organization involved in the collaboration, we had a responsibility to communities to respect their right to not share their data…And that has a lot to do with individual communities’ rights to have governance over their own data.”* (T05 interview)
*“So all of these are sort of contextual factors, having locally placed researchers. In the past, we rely on local people just to get the catering, you know, get the transportation and we want to move beyond that. So the people there [First Nation people] are actually playing a major role in planning research and implementing research.”* (T04 interview)
**Policy**
**3a. Differences in provincial health care systems**
*“There are provincial mandates about how much you can link at one time and how many people…you know system level and bureaucratic challenges but with that each of those provinces sort of have their own primary interests.”* (T08 interview)
*“…trying to understand how primary care physicians are organized and governed in groups…in the three different provinces…very challenging…”* (T02 interview)
*“Some [networks] are situated such that all services and providers are physically located in one building or site. Other are decentralized where a single [network] may have some centralized resources that service more than 35 clinics over a large geographical area. As such, providers are spread out over a large area, and this was a barrier to forming an intervention team…”* (T06 questionnaire)
**3b. Funding models**
*“ None of it would have happened without the ability to…have a team grant and stable funding over time.”* (T12 interview)
*“Having a line in the budget of a region of the province which says, ok, these are the funds devoted to running this program. So as long as this is essentially run on research money, I think the real challenge for implementation, because it does require some additional resources, the challenge is that we want to see results within the same fiscal year, and within the same department.”* (T07 interview)
**3c. Changes in legislation and health care priorities**
“*…there will be change facilitators introduced into academic family practices, there are more allied health professionals that are going to get integrated into those teams…part of the challenge is that we are in the context of a system that is constantly trying to evolve.”* (T07 interview)
*“…that was the change in the responsibility for the [service provider]; to have to take on this home and community care, and so that meant less engagement, you were less well-informed, less well-integrated.”* (T03 interview)
**Multifaceted primary health care context**
**4a. The fragmented structure of PHC**
*“Another weakness is a very low penetration of information technology (IT) to support health care delivery and improved performance. This is a major barrier to the coordination and continuity of care, especially for Canadians with chronic diseases”* (T07 questionnaire).
*“We need to really seriously start to look at technology. Technology does not replace cultural sensitive human contact, but on the other hand, if you try reaching continuity using technology vs. lack of continuity using short term people.”* (T04 interview)
*“I’m always saying that I don’t think that we need a lot of new resources in our health care system, we probably have enough, we just have to start using what we have in a much smarter way. The big issue is integration and organization of these services.”* (T07 interview)
**4b. Competing priorities**
“*…we are not realistic…when we ask busy people to do research there seems to be an assumption that this is their only piece of research that they’re doing but it isn’t.”* (T09 interview)
*“So we had this really key partner who we were working with, and they were probably the number one stakeholder for that group who was most engaged, but then with the influx with refugees, that person was so busy on refugee health care for those people that they pretty well just left the project entirely.”* (T02 interview)

#### Contextual theme I: diversity of jurisdictions

The diversity of settings and jurisdictions that teams worked in influenced the implementation of their innovations. Teams worked across multiple Canadian provinces and territories. Five teams had international partners and three teams worked with Indigenous communities in Canada.

##### Cross-jurisdictional nature of the work

Challenges in working across jurisdictions included differences in provincial structures, limited resources, and maintaining cohesiveness among the research team members.

*“Ensuring cohesiveness among [project] activities and working toward common goals are some of the challenges related to managing a research team involving a large number of players, located in different jurisdictions.”* (T07 questionnaire)

Working across jurisdictions was a barrier to implementing innovations due to the nature of cross-collaborative projects. Ethics approvals were required from multiple jurisdictions and institutions, each with their own set of requirements. Privacy regulations varied across provinces and impacted timely access to data. Working within rural and remote areas with limited access to health care services also impacted innovation implementation.

##### International partnerships

International partnerships promoted collaboration and shared suggestions in development of CBPHC innovations. Collaborating internationally acted both as a facilitator and barrier for shared learning for the teams. Some teams had international research trainees who attempted to replicate Canadian projects to fit their country’s context. Collaborating with international research partners also created challenges due to differences in time zones, research styles, PHC structures and privacy regulations.

*“… the intellectual context in Europe is quite different. So there’s this intellectual exchange that is really important and the other was the notion of how their primary care system works…”* (T11 interview)

##### Multiple languages

Recognizing the need for diversity in languages acted as a facilitator for implementing innovations. The broad term of language was perceived by teams as including unique terminology, cultural differences, and culture of work. Implementing innovations in settings with multiple languages, though challenging, increased the accessibility of the projects across diverse populations. Some teams recognized that concepts could not be easily translated from English into, for example, Indigenous languages.

*“…even the concepts that we were trying to have a dialogue around like health and wellness and well-being and mental wellness. All of those concepts were very difficult to describe in Indigenous languages where the parallel definition did not exist…”* (T05 interview)

#### Contextual theme II: complexity of interactions and collaborations

The success of implementing innovations in CBPHC was dependent on complex collaborations and interactions between research teams, community members, providers, decision makers, patients, and caregivers.

##### Establishing and maintaining relationships

The outcomes of teams’ innovations were dependent on new and existing relationships with diverse partners and stakeholders. Focusing on relationships with others was an enabler for innovation implementation. However, turnover in government officials, partnering decision makers, and clinicians required teams to build new relationships and sometimes modify aspects of their projects.

*“All of those directors have changed, there has been some turnover. Talking about adapting our program to different contexts, well the context keeps changing and we need to keep adapting to their new priorities, new programs and new structure.”* (T07 interview)

Similarly, turnover of clinicians and research personnel sometimes delayed implementation of innovations as new clinical or research staff needed time to be trained.

##### Complexity of being part of the CBPHC 12 teams

There were mixed findings about collaborating across the 12 teams as working within a large cross-jurisdictional network of independent research programs acted both as a barrier and facilitator in implementing innovations. Working across the 12 teams was seen as an opportunity to share ideas, learn about each other’s innovations and increase cross-team collaboration.

*“I think it’s nice to think that you’re part of something bigger and I think that to learn things across 12 teams definitely has the potential to have some powerful outcomes.”* (T06 interview)

One team, for example, adapted an innovation from another team to their own context. The funder’s expectation for the 12 teams to collect common process and outcome indicators, however, was at times recognized as inconsistent with the diversity of the teams in terms of their innovations, the communities and health systems they worked with and the team itself. This was a particularly challenging contextual factor for the teams working with Indigenous communities, where the communities made decisions pertaining to the data that was important to them.

##### Levels of engagement with stakeholders

The level of engagement of stakeholders, such as policy and decision makers as well as clinicians, patients and caregivers, was an important contextual factor that facilitated the successful implementation of innovations. This was often associated with recognizing the potential value of innovations for larger communities.

*“I think in [province] we have achieved some excitement about [innovation] within our health authority…they have invested interest in [innovation], seeing how [innovation] can help inform the work that they’re doing.”* (T02 interview)

Despite the teams’ best efforts in engaging stakeholders, some decision makers still did not “buy-in” to their projects or feel that their projects were worth implementing due to competing priorities and the dynamic nature of the policy worlds.

##### Leadership

Teams described the important contextual factor of leadership to facilitate implementation of innovations and often fostered leadership at various levels (individuals, communities, and organizations) to achieve success. Leadership was perceived by teams as consisting of supportive mentorship and led to trusting relationships among providers in team environments. Strong leadership helped teams to navigate the different perspectives of diverse stakeholders (eg, community members, decision makers, patients and caregivers, researchers). Teams recognized that effective leaders should encourage shared leadership, be open to change, promote and maintain commitment, and ensure that members have the resources to achieve their goals. This made it easier to conduct large CBPHC projects and offset issues that were not always in the control of the teams (eg, time and financial resources).

*“The leadership, the engagement from the beginning and the commitment of the individuals involved in this topic all have led to a more engaged team than you would see in any other projects.”* (T08 interview)

##### Working with Indigenous peoples

Involving Indigenous Peoples in research acted as a facilitator to promote the success of innovations. Recognizing the unique cultural and historical context of Indigenous Peoples in Canada, three teams working with Indigenous communities followed Ownership, Control, Access, and Possession (OCAP®) standards of how to conduct research with First Nations (First Nations Information Governance Centre, 2014). These guidelines assert that First Nations have control over data collection processes in their communities and they own and control how that data will be used.

*“You have to have mutual respect that is spelled out through OCAP® on how to partner and work collaboratively with First Nations. The Truth and Reconciliation [*Truth and Reconciliation Commission of Canada, 2018 *] is a list of recommendations that highlight the issues of colonialism in Canada with creating recommendations as a path to move forward…”* (T01 interview)

When collaborating with Indigenous Peoples, teams also had to navigate complex health care funding structures and limited PHC resources to realize the implementation of innovations in these communities.

#### Contextual theme III: policy

National and provincial political structures and changes to these structures and policies impacted the implementation of CBPHC innovations.

##### Differences in provincial health care systems

The diversity of health care system structures across provinces acted as a barrier to innovation implementation. Researchers had to familiarize themselves with health care systems in different jurisdictions to determine how they could best implement innovations in different settings. These included differences in structure, services, regulations, and interests.

##### Funding models

Teams reported that funding models for PHC services varied within and across provinces, resulting in differences in available health care services, the organization and delivery of health and other social services, and ability to implement innovations across various sectors. Varying funding models acted as barriers to implementation of CBPHC innovations.

*“Funding sources [for usual PHC] were the same in three sites but the funding models differed and, as a result, the services offered were different when comparing acute and community settings.”* (T06 questionnaire)

Teams also discussed the need to create a line in the research budget of the region or province related to the innovation, to ensure sustainability beyond research funds.

##### Changes in legislation and health care priorities

Researchers considered the constant changes in political structures and regulations when implementing innovations. These changes impeded successful implementation of innovations. For example, the requirement of clinicians to meet new practice standards led to lower interest for clinicians and decision makers in supporting this type of research initiatives. Changes in legislation and priorities also led to changes in the organizational resources required for an intervention.

*“…they [health ministry] imposed a quota of patients that practices had to take…well that would definitely influence the practices’ interest in taking part in things, and how we were going to roll out the intervention.”* (T03 interview)

#### Contextual theme IV: the multifaceted nature of PHC

PHC is organized by multiple groups of care providers and stakeholders. Having multiple players involved in PHC has been perceived by the teams to lead to lack of communication with other tiers of service and complex care delivery.

##### The fragmented structure of PHC

Lack of PHC coordination and gaps in services were pronounced both in rural and remote as well as urban areas, influencing innovation implementation. The structure of the current state of PHC acted as a barrier to implementing innovations. The differences in defining PHC across jurisdictions impacted the types of innovations that could be implemented and the evaluation of innovations. The low penetration of information technology in PHC was seen as a particular contextual challenge for the teams.

*“Imagine sort of the fragmentation [of primary care] in larger urban centres. I think that’s sort of a real challenge.”* (T07 interview)

##### Competing priorities

Researchers experienced challenges in implementing innovations when PHC clinicians had to meet the high demands of providing clinical services over research.

*“…we would produce something and then we wouldn’t hear from them [providers]…this is all because of different priorities, right? They have a huge responsibility in front of them and they need to prioritize that, they can’t let care delivery drop.”* (T03 interview)

Researchers also recognized that clinicians were often engaged in a number of different research projects.

### Strategies to address contextual factors

By reflecting on contextual factors that were influential in their work, representatives of the 12 teams also discussed strategies they used to address these factors in order to successfully implement innovations. Six main strategies were identified and are described below. See Table 4 for additional qualitative data.

**Table 4.** Additional examples of data for strategies to address contextual factors in CBPHC

**1. Conducting an environmental scan at the beginning** *“Environmental scans are contextual portraits of [project] communities, that include collating the existing data related to health needs, key healthcare indicators, organization of healthcare services, and available resources and partnerships.”* (T07 questionnaire)
*“…An environmental scan is really sort of the basic part of the design of the program. The first thing that we do, we want to know what’s available. We want to know who are the primary care providers, we want to know about community pharmacies, we want to know about different organizations, different programs, different initiatives that exist within the region because ultimately these are our partners…We don’t create new programs ourselves, but we want to make sure that the existing programs are better utilized.”* (T07 interview)
**2. Maintaining engagement among partners and stakeholders by encouraging open and inclusive communication**
“*We found it necessary to devote significant time engaging stakeholders. Feedback through this engagement influenced our study processes.”* (T02 questionnaire)
*“We committed our partners to be involved and communities to be involved in every aspect of the program and they have been involved in every aspect of the program…it’s not as ideal as we would have hoped for and that is one of the reasons that we are having an October workshop is because we want to ensure that the [First Nation] communities have a full say in the evaluation and knowledge translation phase.”* (T01 interview)
“*We favour open discussions where all [project] investigators and staff are welcome to contribute. As such, we hold weekly teleconferences to provide progress reports, and discuss common opportunities and challenges and higher levels discussion of [project] orientation and strategic planning.”* (T07 questionnaire)
**3. Contextualizing the innovation for different settings**
*“We’re also interested to see if we can go beyond cardiovascular risk factors…In some projects we were also looking at screening for atrial fibrillation because the automated blood pressure devices that we utilize allow us to do that as well. We also looked at the issue of patients who don’t have family physicians…So the interest was to see how the program needs to be again, modified, adapted, and how do we integrate the program with larger primary care?”* (T07 interview)
*“Maybe an important lesson for us is you know the more integration we are trying to do into existing structures, the more changes in context can be a threat. Because you know, we’re relying on more and more things that are outside our control.”* (T03 interview)
*“The idea was that you could demonstrate that it works very well [jurisdiction], we would take it over to [jurisdiction] and say look, your remote system is pretty much the same, why don’t you move to something that is more centralized…”* (T04 interview)
**4. Anticipating and addressing changes, delays, and needing additional resources**
*“So workload, so I was anticipating a certain staffing complement to do these tasks and we pretty much had to double. We pretty much had to have many more staff to do the task to make sure all the patients were being approached in the right way, and that the providers were in the loop and everybody had their responsibility of who they were communicating with.”* (T11 interview)
*“I’m speaking just for the patients’ journey piece right now because you have referenced the people we interviewed and we did some training of students we did sort of a boot-camp to train up students to how to do interviews with children, parents, service provider, policy-makers. So that was another piece that we felt was very important to the project but was very time consuming.”* (T09 interview)
*“…it’s a lot of extra work to be part of the 12 teams and work together….”* (T02 interview)
**5. Fostering a culture of research and innovation among partners and stakeholders**
*“So that is something that I think that unfortunately, research is something that the government says ‘It’s not our job to support research, we will promote it, we will help you facilitate, and write letters of support, but don’t expect us to spend money on’”* (T04 interview)
*“So there is little interest on the part of decision makers who collect any meaningful data. They are usually interested in sort of head count, they are not necessarily interested in sort of the impact, very frequently there is no before data or after data. There is no research culture.”* (T07 interview)
**6. Ensuring information about the innovation is widely available**
*“In the third phase, we aim to translate our research into practice by identifying emerging models of Integrated CBPHC in advance, and working alongside policymakers to inform the development and implementation of these models in each jurisdiction.”* (T10 questionnaire)
*“We’re trying to develop a newsletter, [study member] has been leading that. We have a website, we let people know about publications…”* (T12 interview)
*“…At the mid-way through where we started not just involving our people that’ve experienced any organizations that sort of higher-level discussion. But actually bringing our research outwards to them [organizations], having different kinds of presentations and taking more time to understand their perspectives at each stage of the research process.”* (T08 interview)

1. **Conduct an environmental scan at the beginning**

Some teams conducted environmental scans, scoping and realist reviews, needs assessments and/or reviews at the start of their projects to understand what programs already exist, what works in different contexts, and what constitutes usual care related to the innovation. The evidence provided decision-making stakeholders with program options outside their context-bound purview and focused feedback from PHC providers regarding areas for improvement. Understanding the geographical, political, and social context helped the teams appropriately plan and fine-tune their innovations to better meet the needs of the communities.

*“Our KT group conducted an environmental scan across Canada in terms of what kinds of initiatives have been conducted to improve coordination of care between primary care and [specialists]. So that really allowed us to kind of get a sense of what’s out there and really get input from these different areas in terms of the work that’s already been done.”* (T12 interview)

2. **Maintaining engagement among partners and stakeholders by encouraging open and inclusive communication**

Teams recommended ensuring that all team members, partners, and stakeholders are appropriately engaged and committed throughout the course of their projects. The importance of meaningful engagement was particularly pronounced in the engagement of Indigenous Peoples.

*“So, a lot of our resources went into that for our project to ensure that we had continual engagement of the community. But then I think…let the First Nations take the leadership role in determining some of the questions in the outcome and how things are structured…”* (T05 interview)

To maintain meaningful engagement, teams tailored communication strategies such as workshops and in-person meetings to the particular needs of different partners and stakeholders. These strategies considered how feedback from others would be collected and incorporated into the projects. Teams used various methods of communication such as tele-conferences, newsletters, progress reports, websites and social media, and in-person gatherings.

*“Regular meetings, a collaborative electronic platform and yearly face-to-face meetings have been essential to maintain the momentum for the study and learning from one another.”* (T03 questionnaire)

Unique strategies to address the language context and improve communication across jurisdictions included having a bilingual research team, strong collaborations, investment in communication tools, and focusing on commonalities.

3. **Contextualizing the innovation for different settings**

The teams described how they tested the innovations in different settings and jurisdictions and tailored and adapted the innovation to fit the unique contexts as needed.

*“…by actually testing and modifying the program in different settings, until you sort of demonstrated that yes, you’re going to get positive results, and the program is implementable across different settings, different populations, and of course different diseases.”* (T07 interview)

Integrating feedback from communities and stakeholders was critical in adapting the innovations to local needs. Researchers discussed retaining the core principles of the innovation while tailoring flexible aspects (innovation components and implementation strategies) to ensure effective integration of the innovation into existing care systems.

4. **Anticipating and addressing changes, delays, and the need for additional resources**

Implementing a CBPHC innovation is a very complex process requiring engagement of multiple stakeholders and consideration of many contextual factors. Therefore, research teams recommended planning for changes in implementation plans, flexible timelines, and the need for additional resources.

*“…just thinking about advice for someone who would be taking on this type of project would just be mindful that things will take a lot longer than you may have originally anticipated. It’s probably going to be for the better in the end because there’s going to be different inputs and it’ll lead to hopefully a better outcome.”* (T12 interview)

Teams also had to be responsive to turnover and changes in team composition and expect delays in implementation while having a plan to address these. While collecting feedback and adapting the innovation led to longer implementation timelines, it also led to important program improvements.

5. **Fostering a culture of research and innovation among partners and stakeholders**

Researchers used multiple strategies to foster a culture of innovation among partners and stakeholders. They recognized that research may not be valued in the same ways in practice and policy environments as in academic environments and emphasized the importance of fostering a culture of innovation within health care.

*…we need to think about how can we foster a research culture within our healthcare system?…when we would approach them [providers] we’re considered an outsider rather than seen as part of the team that can help improve patient care. I think that definitely affects uptake of intervention because it’s perceived as not my job, I’m too busy*. (T06 interview)

Many teams were involved with building research capacity among their partners and stakeholders to help facilitate the uptake of innovations. Examples included involving patients and community members in research activities and creating faculty positions in the North.

6. **Ensuring information about the innovation is widely available**

Teams described using numerous strategies to promote awareness and sustainability of the innovation among stakeholders to enhance dissemination of findings. They ensured that information about their innovations was publicly available and developed tailored resources for various stakeholders. This ensured that findings were shared with community members who were not part of the research using social media, websites, policy briefs, newsletters, toolkits and other approaches.

*“We wanted to use it [website] as a tool to disseminate what we were doing and eventually it will be a tool that people will use as a resource and various groups can eventually use it as a resource like teachers, clinicians, families, parents, children, youth…”* (T09 interview)

## Discussion

### Key findings

Based on the perspectives of the 12 teams, implementing CBPHC innovations across jurisdictions is a complex experience involving multiple and interrelated contextual factors and this complexity should be recognized by decision-maker stakeholders. Teams had to frequently adapt their innovations to fit diverse settings. Further, it was challenging for teams to implement innovations in contexts where there were ongoing changes at government levels such as new policies, priorities, structures, and leaders. The 12 teams used a variety of strategies to ensure successful implementation of CBPHC innovations by addressing contextual factors, especially strategies focused on building and maintaining relationships with stakeholders including Indigenous peoples.

### Comparison with the literature

This present study expands on the Tomoaia-Cotisel *et al*. (2013) framework by providing a much more detailed description of contextual factors that impact the implementation of CBPHC innovations. Rather than being limited to five domains of the framework (ie, practice, larger organization, external environment, implementation pathway, and motivation for implementation), our study highlights the interrelatedness and the interactive dynamic of contextual factors in how they mutually influence and transform one another. We also consider the diversity and landscape of jurisdictions as an important factor to consider when implementing innovations across Canada and beyond (Sutherland and Busse, 2016). The current study further extends the work of Tomoaia-Cotisel *et al*. (2013) by providing concrete examples of strategies used by the 12 teams to address contextual factors in implementing PHC innovations.

The contextual factors identified align well with some of the key contextual factors identified by Lau and colleagues (2016) in their review of reviews of complex changes in primary care: (a) professional: attitudes to change; (b) organization: relationships, and processes and systems; and (c) external context: stakeholder buy-in, governance and financing, and policy and legislation. The present study makes a new contribution by highlighting the importance of selected contextual factors, namely, diversity in settings and jurisdictions, and establishing and maintaining relationships with a broad range of stakeholders, including Indigenous peoples. It is critical that PHC innovations take into consideration the contextual challenges faced by Indigenous peoples such as the inadequate patchwork of policies and health care practices currently in existence (Katz *et al*., 2017).

Our results also confirm the conclusions by Lau *et al*. (2016) of the importance of being aware of the dynamic nature of context, as the 12 teams encountered ongoing changes in areas such as decision-maker partners and government priorities and funding. It is not enough to simply assess context at the start of a change project; this needs to be an ongoing effort throughout the phases of implementing the innovation. Teams felt that the poor uptake of information technology was a barrier to health care coordination. There is a need for more innovative approaches in using technology as an instrumental component to practice rather than an electronic documentation system (Gray *et al*., 2018).

The opportunities for shared learning across the 12 teams in this study, and the 14 teams in the study by Tomoaia-Cotisel *et al*. (2013) share some similarities with what has been described as learning communities (Carpenter *et al*., 2018): “a select group of potential adopters and stakeholders who engage in a shared learning process to facilitate adaptation and implementation of innovations” (Carpenter *et al*., 2018: 567). The new incremental learnings generated by the 12 teams have certainly advanced our understanding of the contextual factors important for the implementation of PHC innovations.

### Strengths and limitations

A strength of this evaluation was the inclusion of 12 CBPHC research teams representing diversity in terms of the jurisdictions and settings they involved, the target populations including Indigenous peoples, and the focus of innovation. Two data sources (ie, questionnaires and interviews) contributed to the richness of the data and a rigorous analytic method was used.

However, participants in this evaluation included only researchers and their team members; the inclusion of decision makers, providers, patient and public research partners could have provided different understandings of contextual factors influencing primary care innovation implementation. Such broader stakeholder sampling is recommended for future research. The PHC innovations were focused, as required by the funder, on two key areas, namely, improving access for vulnerable populations, and innovations in chronic disease management and prevention; thus, the contextual factors and strategies to address these factors might be different for other focal areas of PHC.

## Conclusion

This paper provides a comprehensive overview of contextual factors impacting the implementation of CBPHC innovations among 12 multi-jurisdictional teams. Strategies used by the teams to address common contextual factors provide guidance for other research teams interested in implementing CBPHC innovations across different jurisdictions. Research teams should be considering and accommodating context by ensuring flexibility of their programs. Yet funders do not necessarily facilitate such accommodation. Grant requirements and specificities can create rigid barriers for teams to effectively and meaningfully (a) engage different stakeholders (b) develop meaningful partnerships, and (c) achieve results in a dedicated timeframe using specific tools. Future research should explore the relationship between context and outcome measures associated with innovations. Contextual factors also need to be explored in relation to how they impact spread and scale-up of CBPHC innovations. Finally, future research should examine which contextual factors are important, and how, why, when and for whom they are important in various settings, such as the planned realist review on healthcare quality improvement initiatives (Coles *et al*., 2017).

## Supplementary material

For supplementary material accompanying this paper visit http://dx.doi.org/10.1017/S1463423619000483. click here to view supplementary material
