# Supplementary material for: Contextual factors influencing the implementation of innovations in community-based primary health care: the experience of 12 Canadian research teams
Source: Prim Health Care Res Dev. 2019 Jun 28;20:e107. doi: 10.1017/S1463423619000483 (PMC8060818; doi:10.1017/S1463423619000483)
Supplement: Supplementary file 1 [file S1463423619000483sup001.docx]

**Appendix 1. S**ample survey questions

| 1. Briefly describe the project, its background, methods, aims, and expected outcomes. 2. What were the motivations, historical and recent events that contributed to the initiation of this project? (Implementation Pathway and Motivation for Implementation) 3. Describe the project population, their community, norms, and relevant cultural practices. (Practice) 4. Describe the members of the research team. 5. Describe the primary care or community practices involved in the project and their practice setting. (Practice) 6. Describe other stakeholders that were involved in the project and their roles. (Larger Organization) 7. How did the structure of the health care system affect your project? (Larger Organization and External Environment) 8. How did the regional public policy affect your project? (External Environment) 9. What were the unanticipated changes that occurred over the course of the project and what were their impacts? (Implementation Pathway) 10. Describe cross-collaborative and multi-jurisdictional contextual factors encountered in your project and their impact on planning, leadership, and governance? (Larger Organization, External Environment, and Implementation Pathway) |
| --- |

*Note.* The survey questions were developed based on the five Contextual Factor Themes (themes placed in brackets in the table) from: **Tomoaia-Cotisel, A., Scammon, D.L., Waitzman, N.J., Cronholm, P.F., Halladay, J.R., Driscoll, D.L., Solberg, L.I., Hsu, C., Tai-Seale, M., Hiratsuka, V.** and **Shih, S.C.** 2013: Context matters: the experience of 14 research teams in systematically reporting contextual factors important for practice change. *The Annals of Family Medicine*, *11*(supplement 1), S115-S123.

**Appendix 2.** Sample interview questions

| 1. Elaborate on one or two examples of contextual factors that have been most influential in your project. 2. What key contextual factors should be considered if one intends to replicate your project or conduct primary care research? 3. Describe issues and contextual factors encountered while working at a multi-jurisdictional and/or international level. 4. How did differences in the health care system structure and project implementation across multiple provinces affect cross-collaborative efforts? 5. Describe how the diversity of partners affected the design and implementation of your project. 6. Describe your experience in working with a diverse group of stakeholders. 7. Tell us how you promoted effective team collaboration between different groups and perspectives. 8. Describe what you learned from working with community advisory committees and decision-makers. 9. Describe how changes to the health care system impacted your project. 10. Can you describe the impact of policy changes on your project? 11. How, if at all, did being part of the community based primary health care-12 teams impact the design, progression, or outcomes of the project? |
| --- |
